# Identification of a Novel Uromodulin-Like Gene Related to Predator-Induced Bulgy Morph in Anuran Tadpoles by Functional Microarray Analysis

**DOI:** 10.1371/journal.pone.0005936

**Published:** 2009-06-16

**Authors:** Tsukasa Mori, Hiroko Kawachi, Chiharu Imai, Manabu Sugiyama, Youichi Kurata, Osamu Kishida, Kinya Nishimura

**Affiliations:** 1 Laboratory of Marine Molecular Biochemistry, Department of Nihon University College of Bioresource Sciences, Fujisawa, Japan; 2 Graduate School of Fisheries Sciences, Hokkaido University, Hakodate, Japan; Northeastern University, United States of America

## Abstract

Tadpoles of the anuran species *Rana pirica* can undergo predator-specific morphological responses. Exposure to a predation threat by larvae of the salamander *Hynobius retardatus* results in formation of a bulgy body (bulgy morph) with a higher tail. The tadpoles revert to a normal phenotype upon removal of the larval salamander threat. Although predator-induced phenotypic plasticity is of major interest to evolutionary ecologists, the molecular and physiological mechanisms that control this response have yet to be elucidated. In a previous study, we identified various genes that are expressed in the skin of the bulgy morph. However, it proved difficult to determine which of these were key genes in the control of gene expression associated with the bulgy phenotype. Here, we show that a novel gene plays an important role in the phenotypic plasticity producing the bulgy morph. A functional microarray analysis using facial tissue samples of control and bulgy morph tadpoles identified candidate functional genes for predator-specific morphological responses. A larger functional microarray was prepared than in the previous study and used to analyze mRNAs extracted from facial and brain tissues of tadpoles from induction-reversion experiments. We found that a novel uromodulin-like gene, which we name here pirica, was up-regulated and that keratin genes were down-regulated as the period of exposure to larval salamanders increased. Pirica consists of a 1296 bp open reading frame, which is putatively translated into a protein of 432 amino acids. The protein contains a zona pellucida domain similar to that of proteins that function to control water permeability. We found that the gene was expressed in the superficial epidermis of the tadpole skin.

## Introduction

Phenotypic plasticity refers to the ability of a given genotype to produce different phenotypes in distinct environments. Evolutionary biologists have long studied the adaptive significance of phenotypic plasticity of organisms [1], [2] and argued about the conditions that select for plasticity [3], [4]. The genetic basis of phenotypic plasticity continues to be a focal issue for evolutionary ecologists because this knowledge is crucial to understanding its evolution [5]–[7].

Inducible defenses are phenotypic changes induced directly by cues associated with biotic agents and are an example of phenotypic plasticity [8]. Anuran tadpoles have proved to be a useful model organism for studying inducible defenses as they commonly exhibit inducible morphological changes, such as increased tail depth, in the presence of a threat from predators [9], [10]. Previous studies demonstrated that the inducible phenotypic changes in anuran tadpoles have a genetic basis [11], [12], however, we still do not understand how these genetic mechanisms control the morphogenetic changes of inducible defense traits.

Tadpoles of the anuran species, *Rana pirica*, develop a high tail in the presence of dragonfly larvae [13]. Additionally, they can be induced to display another unique morphology, a bulgy body shape, when exposed to a predation threat from their main predator, the larval salamander, *Hynobius retardatus* [14]. The bulgy morph is induced only by the predation threat of the larval salamanders and prevents the tadpoles from being swallowed [13], [14]. *R. pirica* tadpoles are presumed to have evolved the inducible bulgy morphology against the gape-limited *H. retardatus* larvae under an intimate predator-prey relationship [12], [15]–[17]. The induced bulgy morph is not permanent and the tadpoles revert to the normal phenotype when the predation threat is removed [17].

Since *R. pirica* possesses both inducibility and reversibility of the bulgy morph phenotype, it provides a suitable model system for experimental analysis of the genetic basis and mechanism of morphogenetic changes. In a previous study, we performed a cDNA subtraction and microarray analysis using the epithelial tissue of induced and non-induced tadpoles and detected cDNA clones that were uniquely up-regulated or down-regulated in the induced and non-induced tadpoles [18].

Although this report was the first to demonstrate the genetic basis of phenotypic plasticity of the predator-prey interaction by showing different gene expression patterns in bulgy morph and control tadpoles, it did not identify candidate genes related to the bulgy morph. However, the subtraction analysis did identify some transcription factors that are known to be active in brain tissue. It is possible that a higher brain function is involved in predator-induced plasticity in tadpoles because cortisol levels are known to be significantly increased in tadpoles under predation stress (data not shown). Cortisol is a strong indicator of a stress response mediated through the hypothalamic-pituitary-adrenal (HPA)-axis of the vertebrate brain [19].

In a further bid to identify candidate genes that display modified expression patterns associated with this phenotypic plasticity, we prepared a multifunctional cDNA array from a combined sample of facial and brain tissues of normal and bulgy morph tadpoles. Identification of candidate genes will be of value for monitoring up- and down-regulation of genes in the transcriptome of tadpoles during predator-induced formation of the bulgy phenotype and its subsequent reversal.

The microarray analysis identified 32 candidate genes that might be important for controlling the bulgy phenotype. A cluster analysis of the microarray data indicated that only type I and type II-keratin genes, and keratin-related genes were down-regulated in the bulgy morph tadpoles. Keratins are the major structural proteins of the vertebrate epidermis. The analysis also demonstrated that NADH dehydrogenase, aldehyde dehydrogenase, and uromodulin-like genes, including a novel uromodulin-like gene that we name here ‘pirica’, were up-regulated. The uromodulin-like genes represented the largest single category of up-regulated genes, and this type of gene is known to be expressed in epithelial cells. Although aldehyde dehydrogenase genes were also identified by this cluster analysis, their possible role in the genesis of the bulgy morph phenotype is unclear at present. Therefore, we focused our attention on uromodulin-like genes in this study. Uromodulin, also known as Tamm-Horsfall protein (THP), has a zona pellucida domain that may be responsible for polymerization of proteins into filaments and for water permeability [20].

The impermeability of the skin would be useful for retention of water in the tadpole body. Therefore, determination of the location of expression of this novel uromodulin-like gene and whether its predicted protein has a zona pellucida domain will be crucial for interpretation of bulgy body formation in tadpoles.

## Results

### Functional array analysis of bulgy morph and normal tadpoles

**Figure 1:**
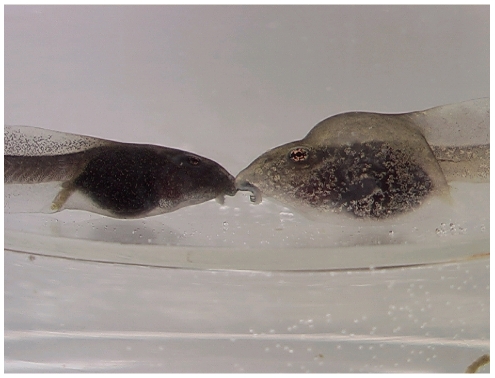
Comparison of tadpoles with a normal phenotype and a bulgy morph phenotype induced by predatory larval salamanders. The normal tadpole is on the left, the bulgy morph tadpole on the right.

**Figure 2:**
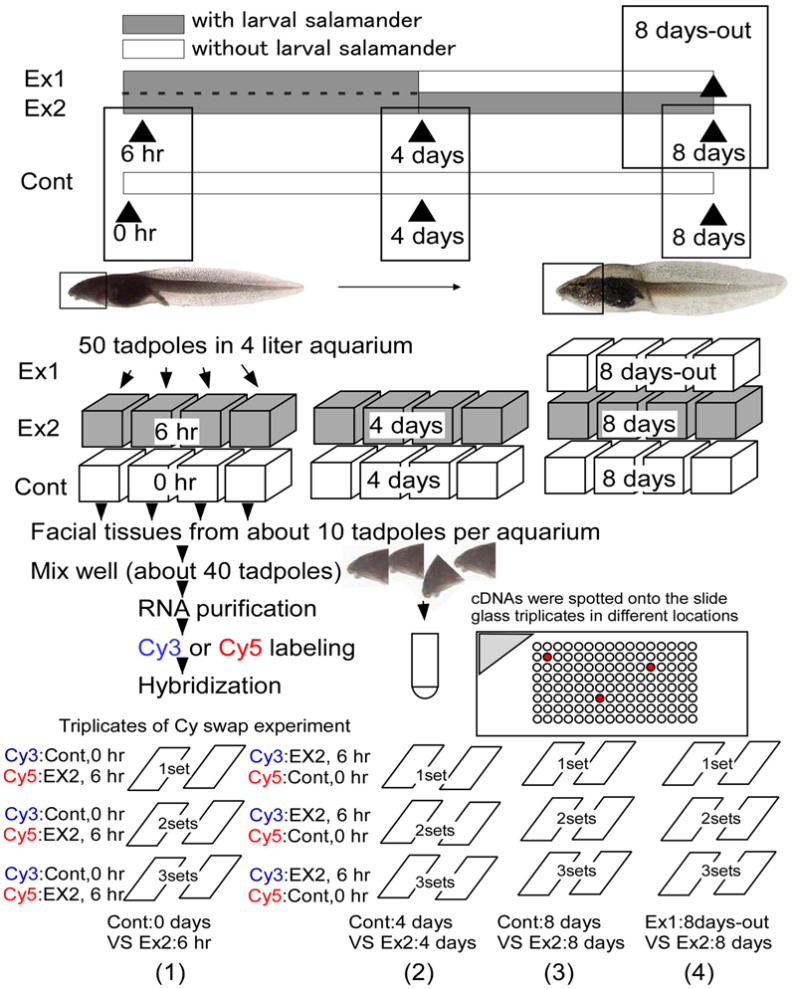
Experimental design used to produce control, bulgy morph and reversion type tadpoles for the functional microarray analysis. One group of tadpoles (Ex1) was placed with a larval salamander for 4 days to induce formation of the bulgy morph phenotype; the predator was then removed and the tadpoles were allowed to revert to the normal phenotype for 4 days. This group is termed “8 days-out tadpoles”. A second group of tadpoles (Ex2) was placed with the predator for the full 8 days. The control group was not exposed to a predator. Tadpoles from the Ex2 groups were sampled at 6 hours, 4 days and 8 days, those from the control group at 0 hour, 4 days, and 8 days. The tissues used for RNA extraction for the functional microarray analysis are indicated by the squares on the tadpoles. The symbols (1) to (4) indicate the comparative design of the microarray analysis, which was performed in triplicate with a dye swap experiment using cDNA microarray spotted onto different locations of the slide glass triplicates.

To identify candidate genes governing the inducible bulgy morph phenotype (Fig. 1), we prepared a functional microarray using 1020 genes selected by subtractive hybridization from 3469 cDNAs (unpublished information). The functional microarray analysis was performed in triplicate with a dye swap experimental design using total RNAs obtained from tadpoles after induction of the bulgy phenotype and during reversion to the normal phenotype (Fig. 2).

Candidate genes for predator-induced phenotypic plasticity were identified by an initial comparison of gene expression profiles in tadpoles with the induced bulgy morph phenotype and controls, and a subsequent comparison with tadpoles undergoing reversion to the normal phenotype following predator removal (Fig. 2).

**Figure 3:**
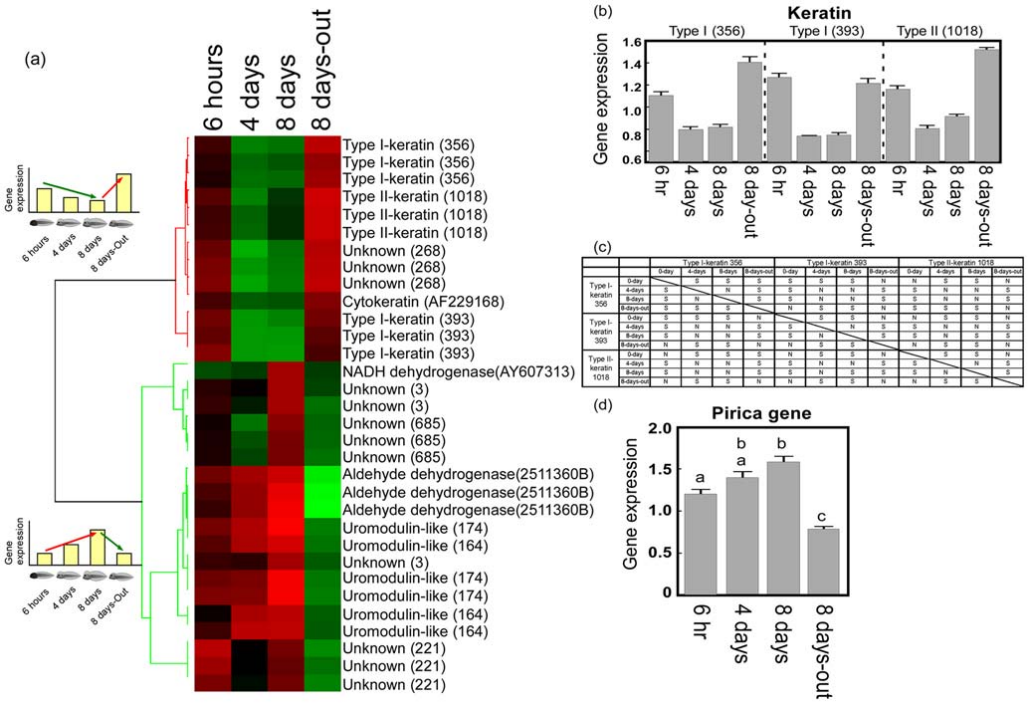
Identification of candidate gene for monitoring up- and down- regulation of genes in the transcriptome of tadpoles using functional microarray analysis. (a) Cluster analysis of microarray data using tissue samples from tadpoles with induced bulgy morph phenotype and those reverting to normal from this phenotype. A Pearson correlation analysis was performed on clusters of up-regulated and down-regulated genes in the two tadpole groups. The columns headed 6 hours, 4 days, 8 days, and 8 days-out were calculated as (Ex2 6 hours/Cont 0 hour), (Ex2 4 days/Cont 4 days), (Ex2 8 days/Cont 8 days), and (Ex1 8 days-out/Ex2 8 days), respectively. Red indicates increased expression compared to median levels of the three replicates with dye swap, whereas green indicates decreased expression. The sequence data for type I-keratin (356), type I-keratin (393), type II-keratin (1018), and the novel uromodulin-like gene, pirica, were deposited in the DNA bank with accession numbers AB374263, AB374264, AB374265, and AB374266, respectively. (b) Relative levels of expression of keratin genes in the microarray analysis. Vertical bars represent standard errors, and the summary of the pairwise comparisons by Dunnett's T3 test is described in (c). The notations ‘S’ and ‘N’ respectively indicate ‘significant’ and ‘not significant’ at the family error rate of 5%. (d) Relative levels of expression of the pirica fragment in the microarray analysis. Vertical bars represent standard errors, and the same letters “a” and “b” indicate no significant differences by Dunnett's T3 multiple comparison post hoc test (p<0.05).

The microarray data (3072 spots) were analyzed using a hierarchical clustering analysis and focusing on two types of gene expression pattern. One pattern was a decrease in gene expression during coexistence with predators and an increase in gene expression when the predators were removed. The second pattern was an increase in gene expression during coexistence with the predators and a decrease in gene expression when the predators were removed (Fig. 3a). Genes that showed significant changes in expression (fold change >1.5, p<0.05) are shown (Fig. 3a); the genes were annotated when the e-value of the blastx or blastn was less than e-30. Type I and type II-keratin and keratin-related genes (cytokeratin) showed the first pattern of expression, i.e. were down-regulated genes. Three unknown genes were also present among the down-regulated group; these genes appeared to be either keratin-like or related to the keratin gene (CAD38126.1, e-value: 1.3). These results indicated that all the genes that showed significant down-regulation during formation of the bulgy phenotype were keratin genes, which are known to encode major structural proteins of the vertebrate epidermis.

A novel uromodulin-like gene, NADH dehydrogenase, and aldehyde dehydrogenase showed the second pattern of expression, i.e. were up-regulated. Uromodulin-like genes were the predominant type showing an up-regulated pattern; uromodulin is also known to be highly expressed in the epidermis. We therefore focused our attention on the genes with known expression in the epidermis, i.e. type I and II-keratins and the novel uromodulin-like gene. The levels of expression of the type I and II-keratin genes and the novel uromodulin-like gene are shown in Figs. 3b, c, and d.

First, we performed a statistical analysis of the three types of keratin gene. One variable analyzed was the level of expression, which depends on two factors: ‘time’ and ‘gene’. The time factor had four groups, ‘0 days’, ‘4 days’, ‘8 days’ and ‘8 days-out’. The genes analyzed were type I-keratin 356, type I-keratin 393, and type II-keratin 1018. A p value less than 0.0005 was obtained using Levene's test for homogeneity of variances, and thus the assumption of homogeneity of the variances was rejected at the 5% significance level. Therefore, an ANOVA is not appropriate, although ANOVA is known to be quite robust where there is inhomogeneity among the variances. We therefore performed a two-way ANOVA and used the results for reference only.

The comparison of type keratin genes were shown in Figs. 3b and 3c. The uromodulin-like genes “uromodulin-like 164” and “uromodulin-like 174” were also analyzed, and indicated that there were no significant differences between these genes. Therefore, they were two fragments of a single gene. A Dunnett's T3 multiple comparison test was used to investigate the time effect after these two genes were combined (Fig. 3d). Details on the processes of statistical analysis for keratins and uromodulin-like genes were described in Statistical analysis for microarray of Materials and Methods.

### Complete sequence of the novel uromodulin-like gene

**Figure 4:**
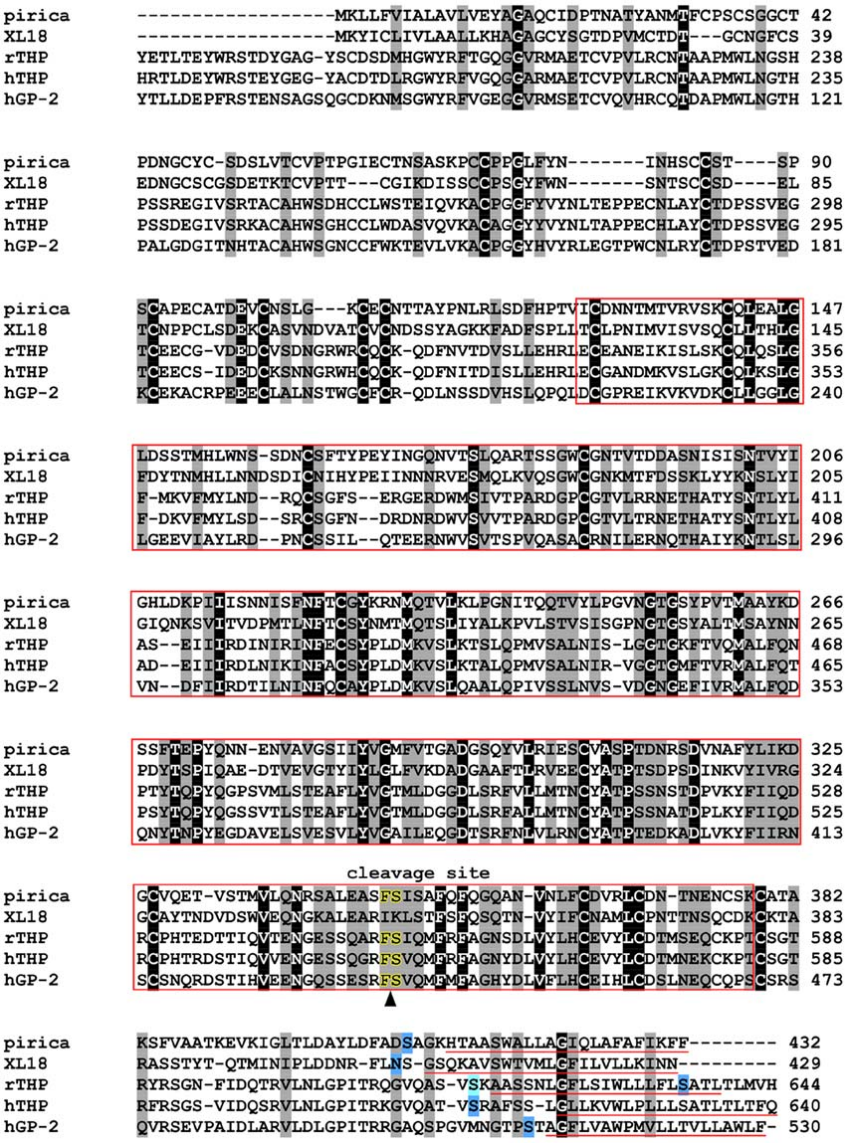
Alignment of the deduced amino acid sequences of the pirica gene with those of homologous/related genes. Conserved residues in all sequences are highlighted: identical, similar and unrelated residues are indicated by dark, light and white backgrounds, respectively. The red box represents the zona pellucida domain. Potential GPI-anchor residues predicted by GPI-SOM (fttp://gpi.unibe.ch/) are represented by black letters with blue background, while those suggested from sequence similarity are highlighted in light blue. Residues underlined in red represent the transmembrane domain predicted by the TMpred program (http://www.ch.embnet.org/). Letters edged in yellow represent the predicted proteolytic cleavage site. All relevant sequences of the uromodulin (THP), GP-2, and XL18 have been registered in the DDBJ/EMBL/GenBank databases with accession numbers XL18 (AAC59871), rTHP (AAB33313), hTHP (P07911), and hGP-2 (AAB19240), respectively.

The complete cDNA of the novel uromodulin-like gene was obtained by 5′- and 3′-RACE using the gene fragments 164 and 174. We found that these two fragments belonged to a single gene, as indicated by the statistical analysis described above, which we name here ‘pirica’. The deduced amino acid sequence of the pirica gene is shown in Fig. 4. The gene consists of a 1296 bp open reading frame, which translates into a protein of 432 amino acid residues. The molecular weight of the protein is estimated at 46 kDa and the pI is calculated as 4.82. The predicted protein has a zona pellucida domain, a C-terminal glycosylphosphatidylinositol (GPI)-anchor sequence, and a proteolytic cleavage site, similar to those that are present in uromodulin (THP) and GP-2 (Fig. 4). A transmembrane domain is also present in pirica. However, human THP (hTHP), and rat THP (rTHP) have predicted proteins of 640 and 644 amino acid residues, and about 30% of the amino acid residues of the EGF domain are absent from the N-terminal region of pirica. This comparison indicated that the pirica gene was a novel uromodulin-like gene.

The pirica gene showed the highest similarity with XL 18 of *Xenopus laevis* with an e-value of 8e-85. The homology of the protein deduced from the cDNA and XL 18 was 41% (identity) and 56% (positive).

The e-values of the pirica gene compared with hTHP, rTHP and human GP-2 (hGP-2) were 6e-31, 2e-25, and 3e-23, respectively. The pirica protein showed 31%, 29% and 26% identity with hTHP, rTHP, and hGP-2, respectively.

### *In situ* hybridization using RNA probes for pirica

**Figure 5:**
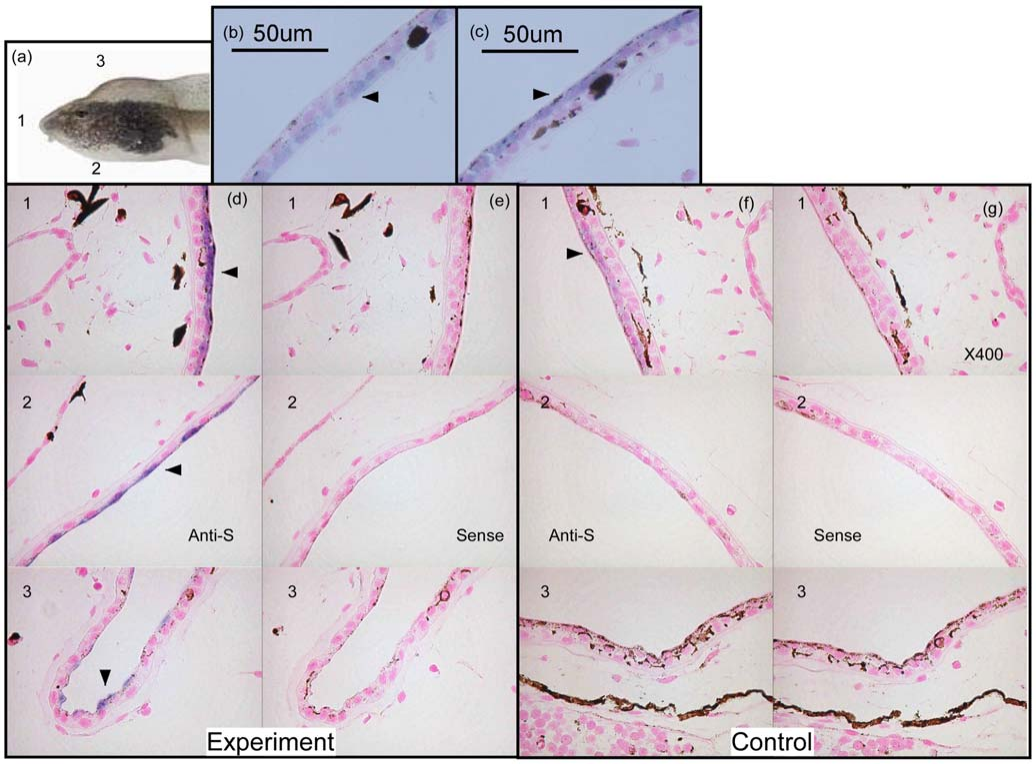
*In situ* hybridization (ISH) analysis of control and bulgy morph tadpoles. (a) Tadpoles were longitudinally sectioned (5 µm) at the frontal side (1), the ventral side (2), and the dorsal side (3). (b) ISH analysis of bulgy tadpoles with an anti-sense probe for type-1 collagen expression. Arrowhead indicates type-1 collagen signal. (c) ISH of bulgy tadpoles with an anti-sense probe for pirica expression. Arrowhead indicates the signal for pirica expression. (d) Pirica gene expression with an anti-sense probe at the frontal side (1), ventral side (2), and dorsal side (3) of a bulgy morph tadpole. (e) ISH of pirica gene expression using a sense probe. (f) ISH of pirica gene expression in a control tadpole using an anti-sense probe. (g) ISH of pirica gene expression in a control tadpole using a sense probe.

Longitudinal sections were cut from tadpoles and the quality of RNA preservation was checked by *in situ* hybridization (ISH) using probes for β-actin and type-1 collagen; clear signals were obtained for both genes. The collagen probe gave a clear signal in the skin of bulgy morph and control tadpoles (Fig. 5b, arrowhead) and was present peripherally in frontal, ventral and dorsal sections.

ISH with an anti-sense RNA probe for pirica gave signals in the outer side of cells that stained with collagen, i.e. the superficial epidermis of the tadpole skin (Fig. 5c). Figures 5b and 5c were obtained from the same bulgy morph tadpole and were adjacent sections (5 µm). Clear pirica signals were observed in frontal, ventral and dorsal sections of the bulgy morph tadpole (Fig. 5d), whereas weak signals were obtained in controls (Fig. 5f). Although signal intensity was far weaker in the control tadpoles, it was still clearly recognizable. In contrast, no signals were obtained in sections of bulgy morph or control tadpoles when a sense RNA probe was used (Fig. 5e, g). Although we demonstrated pirica gene expression in the epidermis of the bulgy morph tadpole, we were not able to obtain any information on whether this gene was expressed in the kidney. There was no evidence for expression of pirica in the brain from ISH on brain tissue sections.

## Discussion

### Bulgy morph and candidate genes

In a previous study, we used subtractive hybridization and microarray analysis to search for genes expressed in the skin of bulgy morph tadpoles induced by predatory larval salamanders [18]. Here, we sought to profile the transcriptome that might be important for predator-induced phenotypic plasticity and therefore used facial plus brain tissue samples for the functional microarray analysis.

Although we identified various genes involved in the formation of the central nerve system using subtractive hybridization, the signal intensity of these genes in the microarray analysis was weak due to the low concentration of mRNA from the brain. However, we also identified genes of importance to formation of the bulgy shape in tadpoles exposed to larval salamanders. A novel uromodulin-like gene, which we named here pirica, was found in this study, and this gene was clearly a strong candidate for involvement in formation of the bulgy morph phenotype on the basis of our further analyses (Figs. 3 and 5). Since the ISH analysis provided no evidence for pirica gene expression in the brain, it is probable that the gene profiled in this experiment is not brain-derived but is produced in the skin and other connective tissues using RNA from facial tissues.

Uromodulin, also known as THP, is an 85 to 95 kDa glycoprotein that is synthesized by the kidney epithelial cells of all placental mammals [21], [22]. Although, uromodulin is the most abundant urinary protein, its function remains unclear. Uromodulin may play a critical role in some aspects of urinary physiology. First, it may have an immunoregulatory function as it binds interleukin (IL)-1, IL-2, tumor necrosis factor, complement 1q, and immunoglobulins and inhibits lectin- and IL-induced T-cell activation [23]–[26]. Second, there is evidence that uromodulin is involved in regulating urinary stone formation [27]. Third, uromodulin may be a defensive factor in the urinary tract against uropathogenic *E. coli* infection stimulated by high concentrations of mannose residues. Uromodulin can bind to type-1 fimbriated *E coli*, the most common pathogen causing urinary tract infections [28], [29]. Fourth, uromodulin has a gel-forming capability that may influence water permeability as described below.

Uromodulin has a zona pellucida domain, an N-terminal signal sequence, and a putative C-terminal GPI anchor sequence [30]; these important peptide motifs are also present in the uromodulin proteins XL-18 and hGP-2, and in pirica (Fig. 4). GPI-anchored proteins normally attach to the outer surface of the plasma membrane [31], [32]. GP-2 and uromodulin have been shown to be expressed in the apical secretory compartments of polarized epithelial cells within the pancreas and kidney, respectively [33], [34]. The GP-2 and uromodulin family, a new class of GPI-anchored proteins, is likely to play a critical role in intracellular membrane assembly, regulated protein secretion and ion transport [35], [36]. Although pirica is highly homologous to XL18, there is no deduced proteolytic cleavage site in XL 18 between F (Phe) and S (Ser) upstream of the possible GPI-anchor attachment, as is present in the other uromodulin proteins [37] (Fig. 4). The soluble ectodomains of GP-2 and uromodulin are actively released from membranes by proteolytic cleavage, and are present in pancreatic fluid and urine, respectively [37]. This may imply that XL18 and pirica have slightly different functions although they do appear to be very similar types of gene.

Uromodulin has been shown to be localized in the thick ascending limb of Henle's loop (TALH) and in the early distal convoluted tubules of the kidney of placental mammals [38], [39]. TALH is the diluting segment of the mammalian kidney and absorbs Na and Cl ions but not water, allowing the kidney to pass dilute urine in the absence of an antidiuretic hormone that might affect the permeability of the collecting ducts [40].

The C-terminal zona pellucida domain of uromodulin enables the protein to polymerize into filaments [20] and facilitates its gel-forming mucoid capability [41]. It has been suggested that the gel-forming capability of uromodulin within the TALH may contribute to the water permeability of the nephron [22]. An *in vitro* experiment using isolated uromodulin demonstrated that it could act as a water barrier but that it allowed ion movement [42].

Recently, an immunocytochemical study found that uromodulin was present in the kidney and skin of the frog *Rana temporaria* and that uromodulin-positive material was present in the distal renal tubules and nephric ducts of frogs, and in the superficial epidermis of the skin [43]. Amphibia produce urine that is more dilute than plasma and this is a function of the distal tubule of the mesonephros, or the diluting segment, which resembles the TALH in function [44]. Frog skin also resembles the diluting segment of the kidney in that it can transport Na and Cl ions [45], [46]. These data suggest that uromodulin, or a functionally related protein to that observed in placental mammals, is present in frogs. Various bird and reptile species that were screened for an immunoreaction to an antibody against human uromodulin showed no reactivity. However, the superficial layers of the skin of several amphibians and fish, the superficial layers of the oral mucosa and gills of fish, and the distal tubules of the kidney of some amphibians, reacted with the antibody [47]. These data suggest that uromodulin appeared early in invertebrate phylogeny, and its tissue distribution is consistent with the hypothesis that uromodulin functions in regulating water permeability.

The osmoregulatory system of the tadpole involves three organs, namely the gut, kidney and gills (similarly to freshwater fish), while in adult frogs four tissues are involved, namely the gut, kidney, urinary bladder and skin. In adults, cutaneous permeability results in substantial evaporative water loss when the animals are out of the water; this loss is compensated by water re-absorption through the nephron and the urinary bladder and, when the animals return to water, by uptake through the skin [48]. Therefore, formation of the bulgy shape in tadpoles may require alteration of the water permeability of the superficial epidermis of tadpole skin that might be achieved by expression of pirica (Fig. 5). Water retention in the tadpole body would thus use water re-absorbed through the nephron and the urinary bladder.

We found that type I and II-keratin genes were down-regulated in the bulgy morph phenotype. Keratins are the major structural proteins of the vertebrate epidermis, and mutations that perturb keratin filament assembly *in vitro* can cause blistering skin disorders *in vivo* [49]. Keratin filaments impart mechanical strength to keratinocytes, without which the cells become fragile and prone to rupturing upon physical stress [50]. Keratin intermediate filaments also have a role in maintenance of cell shape against osmotic stress [51]. Therefore, down-regulation of keratin gene expression might be necessary for flexibility in tadpole shape and also might increase the available space for water retention, as has been reported for blistering skin disorders resulting from keratin mutation [49], or by cell rupturing due to physical stress [50]. Coordination of expression of pirica and type I and II-keratin genes might be required to initiate rapid formation of the bulgy morph phenotype.

## Materials and Methods

### RNA isolation for probe of functional microarray from tadpoles

The facial segment, including the brain, was dissected from each tadpole, cut into small pieces, and collected together in a single tube containing RNAlater™ (QIAGEN RNA stabilization reagent) for subsequent use in producing RNA probes for the functional microarray analysis. The tissues were stored at −80°C until RNA extraction. Total RNA was obtained using the Qiagen RNA medium preparation system. RNA yield was measured by absorbance at 260 nm and the quality was assessed by confirming the integrity of the rRNA bands by electrophoresis.

### Subtractive hybridization, TA cloning, and DNA sequencing

We applied a subtractive hybridization strategy using tissues from bulgy morph and normal tadpoles (head skin, face, and the body segment containing the internal organs) to produce a cDNA population enriched in genes controlling the bulgy morph phenotype induced by predatory larval salamanders. cDNA synthesis and subtraction were performed using PCR-Select cDNA Subtraction Kits (Clontech) following the manufacturer's instructions. To obtain genes stimulated by the larval salamander, fully bulgy form of tadpoles induced by larval salamander and controls (about thirty each) were obtained by the former method of Mori et al.(2005) [18]. Purified mRNA from the head skin, face, and part containing the internal organs from bulgy tadpoles induced by larval salamander was used as the tester, and mRNA from those parts of control tadpoles was used as a driver. To obtain genes suppressed due to predation threat by larval salamander, mRNA from the head skin, face, and part containing the internal organs from control tadpoles was used as the tester, and the mRNA from those of bulgy tadpoles was used as a driver. Tester and driver cDNAs were separately digested with *Rsa* I. The *Rsa* I-digested tester cDNA was ligated with adapters and hybridized with an excess amount of *Rsa* I-digested driver cDNA to subtract the cDNA population present in both tester and driver. Residual single-stranded cDNA, rich in tester-specific cDNA population, was specifically amplified by PCR with primers corresponding to the adapters. Subtracted PCR products (4 µl) were mixed with pCRII-TOPO (Invitrogen) solution (1 µl of diluted salt solution to 1/4 with DW, 1 µl of vector), and incubated for 5 min at room temperature. The mixture (2 µl) was transferred to an electro cuvette containing 50 µl of *Escherichia coli* (Top10; prepared for an electroporation) and transformed into the bacteria using Micro-Pulser (Bio-Rad). Transformed cells were cultured on LB agar plates supplemented with ampicillin (50 ppm), X-gal and IPTG, and insert-containing colonies were selected by blue-white selection. Single colonies were picked and screened for inserts using PCR. Randomly picked clones were sequenced for inserts using M13 Reverse or M13 (−20) Forward primers. DNA sequencing reaction was performed using BigDye Terminator v3.1 Cycle Sequencing kits (Applied Biosystems), and sequencing samples were analyzed on ABI 3100 DNA sequencer (Applied Biosystems). Sequences were submitted for blastn and blastx analysis using public domain DDBJ databases (http://blast.ddbj.nig.ac.jp/top-e.html), and search homology through cDNA and protein was used. Annotation was assigned through the use of protein homology database using homologous sequences and clones were categorized as known or novel gene. Selected 1020 genes were used for functional cDNA chip from subtracted 3469 cDNA.

### Preparation of functional cDNA microarray chip

Inserts in the pCRII-TOPO vector (Invitrogen) from the subtraction experiments were amplified with PCR using primers from sequences flanking the cloning site. The PCR products (1020 clones) were visualized on a 0.8% agarose gel to ensure adequate PCR amplification prior to being robotically printed onto glass slides (DNA Chip Research Inc). After electrophoresis, PCR products were precipitated using isopropanol without salt and glycogen. These PCR products were spotted onto the slide glass triplicates, and chalcone isomerase (CHI) and dihydroflavonol 4-reductase (DFR) from a *Lotus japonicus* cDNA clone were used as external positive controls. λDNA and pCRII-TOPO vector without an insert were used as external negative controls, and these negative and positive controls were used for checking the DNA chip quality and hybridization efficiency. Total RNAs (5 µg) were extracted from the facial parts of bulgy morph and control tadpoles in the various experiments, and double strand cDNA was synthesized using MessageAmpII aRNA Amplification Kit (Ambion). Then, *in vitro* transcription to synthesize aminoallyl-labeled anti-sense RNA was performed using MessageAmpII aRNA Amplification Kit. The synthesized aminoallyl-labeled anti-sense RNAs (8 µg) were coupled with Cy5 or Cy3 using a Mono-Reactive Dye Pack (GE Healthcare) according to the manufacturer's instructions. In brief, the positive control (CHI and DFR) genes were inserted into pT7Blue (Novagen) and amplified with the T7 primer (5′-CAAGCTCTAATACGACTCACTATA GG-3′) and the U-19 primer fused with oligo-dT25 primer (5′-T_(25)_GTTTTCCCA GTCACGACGT-3′). Anti-sense RNAs from positive controls were synthesized from these PCR products using a T7 RNA Synthesis Kit (Wako). These anti-sense RNAs from positive controls were used for synthesis of aminoallyl-labeled anti-sense RNAs as described above. The CHI (5 ng) and DFR (6 ng) aminoallyl-labeled anti-sense RNAs (positive controls) were each added to 20 µg of RNA (bulgy tadpole or control). The mixtures of aminoallyl-labeled anti-sense RNAs were precipitated with ethanol, the pellets dried, and then redissolved in 19 µl of coupling buffer (MessageAmpII aRNA Amplification Kit). The aminoallyl anti-sense RNAs (9 µl) were then coupled with either 11 µl of diluted Cy3 or Cy5 with DMSO using Mono-Reactive Dye Pack. Then, 4.5 µl of hydroxyl amine provided in the kit (4 M) was added to the Cys labeled aminoallyl anti-sense RNAs (20 µl) and incubated at room temperature for 15 min in the dark to quench unlabeled free Cys. RNase free water (5.5 µl) was added to the mixture, and Cys labeled anti-sense RNA was purified (20 µl) using the column provided in the kit. These labeled aminoallyl anti-sense RNAs were electrophoresed on a 1% gel, and the labeling efficiency was determined using Image Quant software (GE Healthcare) after scanning the gel with a Typhoon 8600 (GE Healthcare). After checking aminoallyl anti-sense RNA labeling, these samples were used as Cys anti-sense RNA hybridization probes.

A dye swap experimental design comparing the same samples but using opposite dyes in the two hybridizations was used to compensate for dye-specific labeling effects. Cy3 and Cy5 labeled anti-sense RNA (15 µl) solutions were mixed and supplemented with 12.5 µl 20×SSC (3 M NaCl, 0.3 M C_6_H_5_Na_3_O_7_·2H_2_O), 2.5 µl 10% SDS. The solution was heated at 95°C for 2 min, and then incubated on ice for 30 seconds. Formamide (5 µl) was added to the mixture to form the hybridization solution, which was incubated at 42°C for 5 min. The pre-warmed hybridization solution was dropped onto DNA chip slides, and a coverglass was placed over the solution on each slide without forming bubbles. The DNA chips were incubated in an ArrayIt Hybridization Cassette (Telechem International, Inc.), and hybridized at 65°C for 16 hours. After hybridization, DNA chips were soaked in 2×SSC, 0.1% SDS solution at room temperature, and coverglasses removed by a gentle rocking of the slides. DNA chips were washed in the 2×SSC, 0.1% SDS solution for 20 min, with occasional gentle shaking. Chips were subsequently soaked in low stringency wash buffers (0.2×SSC, 0.1% SDS solution) and (0.05×SSC, 0.1% SDS solution) for 20 min each. The chips were then dried by centrifugation at 600 rpm for 2 min, and scanned using a GenePix 4100A (Axon Instruments) with an integrated Cy5/Cy3 count ratio of 1.0. The data were analyzed using GenePix Pro 6.0 software (Axon Instruments) according to the manufacturer's manuals.

All microarray data have been deposited in Gene Expression Omnibus (GEO) (http://www.ncbi.nlm.nih.gov/geo/), platform accession number GPL6976. Sample accession numbers are GSM299467 to GSM303324, and series accession number is GSE 12005.

### Experimental design

Eggs of *R. pirica* and *H. retardatus* were collected from a pond in Hokkaido, Japan, and placed in 12-liter aquaria. After hatching, *R. pirica* tadpoles were fed rabbit chow ad libitum. The larval *H. retardatus* were fed small-sized *R. pirica* tadpoles ad libitum. Water in all aquaria was changed every second day. The experiment was conducted in a laboratory at 18°C, using a natural day/night (about 14/10 hours) regime. The experimental units were 4 liter aquaria (29×16.5 cm in surface area, and 9 cm in height) each filled with 2 liters of aged tap water. Fifty similarly sized 10-day-old tadpoles (mean±standard deviation: body length  =  7.90±0.38 mm, n  =  48) were randomly chosen from the holding tank, and were placed in each aquarium. The tadpoles were fed rabbit chow ad libitum daily, and the water of all aquaria was changed every second day throughout the experiment. The experiment consists of two treatments, predator-treatment (Ex1 and Ex2) and control (Cont), each of which had 20 (including backup) and 12 aquaria, respectively. The experiment was started when three larval salamanders were introduced in each predator-treatment (Ex1 and Ex2) aquarium (their size was about 18 mm). At 6 hours and 4 days after starting the experiment, we randomly choose 4 aquaria in each treatment and sampled the tadpoles in the aquaria, respectively. After 4-day sampling, we removed the predators from the remaining aquaria of the predator-treatment to make a response of phenotypic revision of the bulgy tadpoles (Ex1). At 8 days, we sampled the tadpoles in the aquaria of each treatment.

During the experiment, to minimize unexpected predation of tadpoles in the predator-treatment aquarium, the salamander was replaced daily with another that had been kept in a holding tank containing sufficient *R. pirica* tadpoles to allow easy feeding. Replacement predators were randomly chosen from each holding tank. Every second day, we counted the tadpoles in each aquarium to check the number of survivors. If unexpected deaths reduced the number of tadpoles in an aquarium, we reallocated tadpoles from another aquarium, which was chosen arbitrarily, to maintain a minimum of 50 tadpoles per aquarium in each treatment. Through such manipulation, we sought to eliminate any possible density effect on experimental results. Since it was not practical to make the experiment sufficiently large to encompass all possible variance components in the induction-reversion experiment that might influence the functional microarray analysis, the sampling protocol in our experimental design ignores any inter-aquaria variation and between individual tadpole variation.

For the microarray, RNAs were extracted and prepared at each sampling time from four sets of fifty tadpoles from the 4 liter aquaria. Each aquarium was used only once at each sampling time and approximately ten tadpoles were harvested from each aquarium. Facial tissue (including the brain) was dissected from the tadpoles and cut into small pieces; the samples from the four aquaria of each treatment group were mixed and used for the RNA extractions. The extracted RNA from each group of 40 tadpoles served as replications in each comparative experiment. Microarray hybridization was performed as described in Fig. 2. The symbols (1) to (4) in Fig. 2 indicate the comparative design of the microarray analysis, which was performed in triplicate with a dye swap experiment using cDNA microarray spotted onto different locations of the slide glass triplicates. As each experiment was performed in triplicate using 6 different cDNA chips, a total of 24 cDNA chips for the whole experiment.

### Statistical analysis for microarray

It is not possible to detect important genes in a very large microarray by statistical analysis only without also obtaining false positives. Although the Bonferroni analysis can detect genes without false positives, in general it is regarded as being too restrictive in its selection of genes and its use can result in the failure to detect any significant genes or the loss of many extremely important genes. The preferred approach is to select genes using a loose statistical restriction together with knowledge of the relevant biology of the experimental system, and then to eliminate false positives by real-time PCR or ISH. We adopted the latter strategy in our microarray analysis, and the selected genes, such as uromodulin-like and keratin genes, were subjected to post hoc multiple comparison tests. Finally, a novel uromodulin-like gene was investigated by ISH to eliminate the possibility of a false positive in the microarray analysis.

Background signals of the microarray were subtracted from the raw data, and then these data were subjected to Lowess normalization (locally weighted scatter plot smoother) between Cy5 and Cy3. Signal intensities under 1000 were eliminated. The average signal intensities of Cy3 and Cy5 were transformed into Cy5/Cy3 ratios. These ratios were further transformed into log ratios and then dye swap log ratios (experimental/control) were produced. These transformed dye swap log ratios were analyzed using an unpaired Student's t-test (fold change >1.5, p<0.05) to order the up-regulated and down-regulated genes according to the time series. A hierarchical clustering algorithm was performed using Avadis 4.3 software (Strand Inc.) to identify complete linkage from significant data obtained from 8 days versus 8 days-out; similarities between gene expression data were measured by a Pearson Centered correlation. After identifying candidate genes using the hierarchical clustering algorithm, we selected the uromodulin-like and keratin genes as being particularly relevant because of their physiological roles and then performed statistical analyses on these selected genes. Although the experimental design allowed use of a two-way analysis of variance (ANOVA), a Levene's multiple comparison test for variances indicated that the assumption of homogeneity of variances was rejected at the 5% significance level. We therefore used the two-way ANOVA for reference only, since ANOVA is quite, but not entirely, robust to departure from the assumption for homogeneity of variance (see Box (1954) [52] and Rogan and Keselman (1977) [53]). For our statistical decisions, we used Dunnett's T3 multiple comparison test for means which is robust for testing means under heterogeneous variance (see Dunnett (1980) [54]).

According to this ANOVA of the three types of keratin gene, all the main effects and interaction effects were significant at the 5% level; p values for the main effects due to ‘time’ and ‘gene’ were less than 0.0005 and 0.003, respectively, and for the interaction of 0.001. The result of a two tailed test is equivalent to that of a confidence interval with the same significance level or confidence. For example, the rejection region of the two tailed test for population mean with 5% significance level is equivalent to the outside region of a 95% confidence interval for the mean. We carried out a post hoc Dunnett's T3 pairwise multiple comparison test on the interaction effect due to ‘time’ and ‘gene’. The 95% confidence interval for the population mean level of gene expression at (‘type I-keratin 356’, ‘0 days’) minus that at (‘type I-keratin 356’, ‘4 days’) was (0.1078, 0.4947). Therefore, the difference between the population mean level of gene expression at (type I-keratin 356, 0 days) and that at (type I-keratin 356, 4 days) was significant at the familywise error rate of 5%. The 95% confidence interval for the population mean level of gene expression at (type I-keratin 356, 0 days) minus that at (type I-keratin 393, 8 days-out) was (−0.2911, 0.0615). Therefore, the difference between the population mean level at (type I-keratin 356, 0 days) and that at (type I-keratin 393, 8 days-out) was not significant at the familywise error rate of 5%. The mean levels of gene expression at 0 days were significantly different between type I-keratin 356 and type I-keratin 393, but the differences between these two genes were not significant at the other time intervals. A similar comparison for type I-keratin 356 and type II-keratin 1018 showed that there was no significant difference in the levels of expression between these genes at any time interval. The comparison of type I-keratin 393 and type II-keratin 1018 showed that the mean levels of gene expression at 8 days were significantly different, but that there were no significant differences at any other time interval (Fig. 3b and 3c).

The uromodulin-like genes, ‘uromodulin-like 164’ and ‘uromodulin-like 174’, were also analyzed in a similar fashion as above. The Levene's test for homogeneity of variances gave a p value less than 0.0005, and we therefore performed a two-way ANOVA for reference only. All the main effects and interaction effects were significant at the 5% level: p values for the main effects due to ‘time’ and ‘gene’ were less than 0.0005 and 0.42, respectively, and for the interaction of 0.095. These analyses indicated that there were no significant differences between uromodulin-like 164 and uromodulin-like 174, and therefore, that they were two fragments of a single gene. Therefore, the data for these two genes were combined, and a Dunnett's T3 multiple comparison test was used to investigate the time effect (Fig. 3d). The expression of the uromodulin-like gene increased significantly (p<0.05) at 8 days compared to 6 hr, but was significantly lower (p<0.05) at 8 days compared to 8 days-out.
